# Identification, sexual dimorphism and aspects of the natural history of *Sapria himalayana* (Rafflesiaceae) on Vietnam’s Lang Biang Plateau

**DOI:** 10.1186/s40529-018-0243-9

**Published:** 2018-12-10

**Authors:** Hữu Đăng Trần, Hồng Trường Lưu, Quốc Đạt Nguyễn, Hiếu Cường Nguyễn, Parusuraman Athen, K. M. Wong

**Affiliations:** 1 Southern Institute of Ecology, Vietnam Academy of Science and Technology, 1 Mạc Đĩnh Chi Street, District 1, Ho Chi Minh City, Vietnam; 2 Graduate University of Science and Technology, Vietnam Academy of Science and Technology, 18 Hoàng Quốc Việt, Cầu Giấy, Hanoi, Vietnam; 3 Singapore Botanic Gardens, National Parks Board, 1 Cluny Road, Singapore, 259569 Singapore

**Keywords:** Da Lat, Lang Biang Plateau, Morphology, Parasitic plants, *Sapria himalayana*, Sexual dimorphism

## Abstract

**Background:**

*Sapria* is a distinctive and narrowly host-specific holoparasitic genus belonging to the Rafflesiaceae. *Sapria himalayana*, rare throughout its range from NE India, SW China, Thailand to Vietnam, is a little-understood species first recorded for Vietnam in 1959, and only recollected there over half a century later in February 2017. This has facilitated an assessment of its taxonomic identity and our understanding of its morphology and natural history aspects.

**Results:**

Six populations of *Sapria* at Vietnam’s Tuyền Lâm Lake, and another two populations at the Nam Ban Protection Forest and the Cam Ly area were found, in an area of about 20 km in radius. Previously documented size attributes, morphological details and colour patterns allowed clear identification of the Vietnamese taxon as *Sapria himalayana* f. *albovinosa*. A full description of the species for Vietnam is provided. Past authors have distinguished the sexes by column form and structure, colour of the upper disk, details of the inner surface of the perigone tube, and presence of ovarial chambers below the column in the female. We present additional observations that male flowers consistently have more steeply held perigone lobes than females, in which the lobes were more spread out at wider angles in fully open flowers, and that males have a much lower cupule than females. The latter difference, especially, appears to be useful for quick determination of the sex even in the advanced floral bud stage. The host plant was the lianescent *Tetrastigma laoticum* (Vitaceae), but superficially it was not possible to ascertain the clonal relationship of neighbouring host lianas. Male and female flowers were found mixed together in the same cluster from one individual liana. Potential pollinators included Calliphorid and Stratiomyid flies observed visiting open flowers.

**Conclusions:**

Our observations have added to an increased understanding of the morphology of this highly specialized parasitic life form. More than this, we have ascertained its occurrence in Vietnam, with information made available to authorities of the Lâm Đồng Province where our studies were conducted, for the sites to be specially demarcated for conservation and carefully managed tourism use.

## Background

*Sapria* Griff. is one of three genera belonging to the Rafflesiaceae, in which all members are holoparasites on various species of *Tetrastigma* (Vitaceae) lianas. They are all narrowly host-specific and produce either male or female flowers. The most conspicuous part of the saucer-shaped open flower is the perianth complement of fleshy lobes (also known as the perigone lobes) that spread out and recurve, and which are frequently deep red to orange. *Sapria* is well distinguished from the other two genera (Hansen 1973; Meijer 1997). *Rafflesia* R.Br. ex Thomson has 5(−6) imbricate perigone lobes in the flower, *Rhizanthes* Dumort. has 16–18 valvate lobes, but *Sapria* has 10 valvate lobes separated as an outer and an inner series. In *Rhizanthes*, the perianth (or perigone) tube does not develop any membraneous outgrowth (‘diaphragm’) just below the base of the perigone lobes, which have a smooth inner surface, but in the other two genera the flower develops a broad diaphragm that defines a central aperture and the perigone lobes are conspicuously warty on the inner side. Whereas perigone lobes are rounded in both *Rafflesia* and *Sapria*, those in *Rhizanthes* terminate in an abruptly attenuated bayonet-like apex. Also, small, irregularly knob-, finger-, or tube-shaped fleshy outgrowths develop on the inner perigone tube surface in *Rafflesia*, but these are found on the diaphragm in *Sapria*. *Sapria* presently consists of three recognized species: *S. himalayana* Griff. (from NE India, SW China, Thailand to Vietnam), *S. poilanei* Gagnep. (Cambodia and Thailand) and *S. ram* Bänziger and Hansen (Bänziger et al. 1997, 2000) (found only in Thailand).

**Fig. 1:**
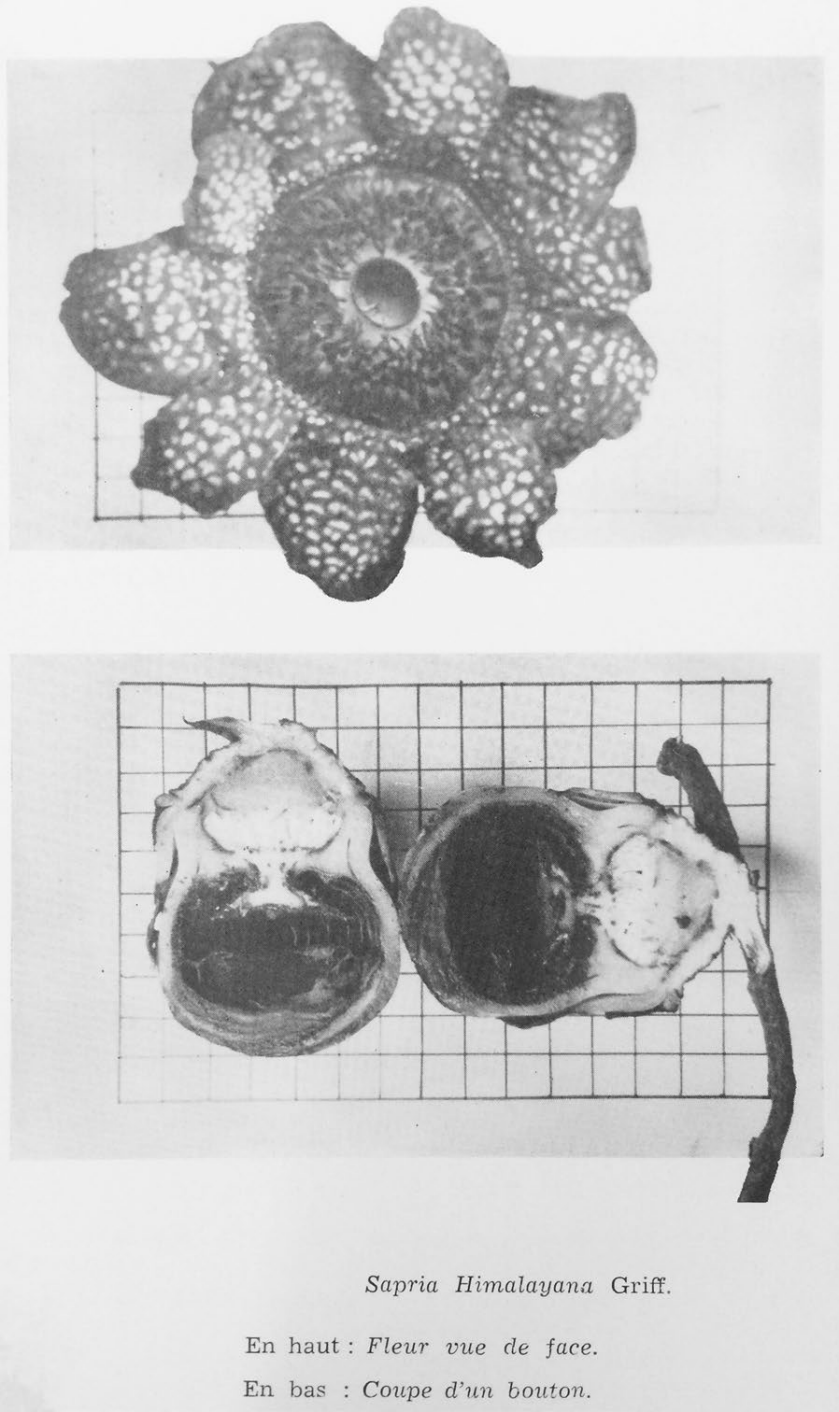
First ever collection of *Sapria himalayana* Griff. (Tixier s.n.) for Vietnam from the Lang Biang Plateau, as photographed in Tixier (1959), showing female flower in top view (above) and young female flower bud in longitudinal section (below)

*Sapria himalayana* was first recorded on the Lang Biang Plateau in Vietnam by Tixier (1959) (Fig. 1). Since then, there have been no other collections made for *S. himalayana* in Vietnam. Subsequent mention of *Sapria himalayana* from Vietnam in the literature (Hansen 1973; Schmid 1974; Pham-hoang 2003) were all based on Tixier’s collection. Unfortunately, we could not locate where the specimens were deposited.

In early February 2016, a picture of a beautiful, red, huge, geophytic flower was sent to the first author by a relative from Lang Biang Plateau. Although it was identified as *Sapria himalayana*, no proper collections were made. In February 2017, during a botanical survey in Nam Ban Protection Forest, Lâm Đồng province by the Southern Institute of Ecology, and also during a field trip to Đà Lạt by the first and last authors, several populations of *Sapria himalayana* were again discovered in bloom, and it was then possible to make appropriate botanical specimens.

The recent rediscovery of *Sapria himalayana* populations on Vietnam’s Lang Biang Plateau offered the opportunity to verify if it belongs to one of the known forms of Bänziger et al. (2000), whose work has dealt with the morphological variation in some detail with excellent information on the distinction of the two forms that they recognised.

## Methods and materials

The surveys resulted in the documentation of six populations of *Sapria* (of which two had open flowers) at Tuyền Lâm Lake, and another two populations with open flowers at the Nam Ban Protection Forest and the Cam Ly area. They all occur in a region of about 20 km in radius.

**Fig. 2:**
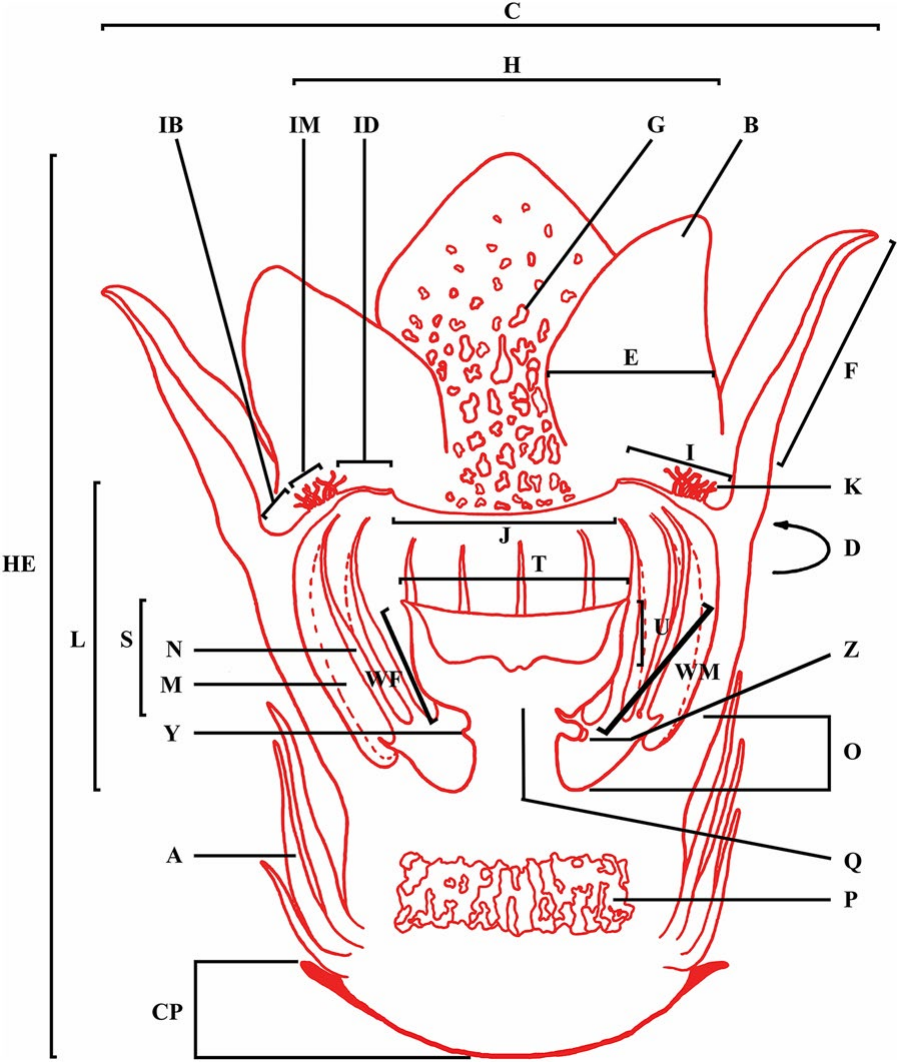
Cross section of the *Sapria* flower, with morphological parts referred to. A. Basal bracts. B. Perigone lobe. C. Flower diameter. CP. Height of cupule at flower base. D. Circumference of perigone tube at lobe base. E. Perigone lobe width. F. Perigone lobe length. G. Wart. H. Diameter of whole diaphragm. HE. Height of flower. I. Width of diaphragm collar. IB. Basal band. ID. Width of distal band of diaphragm. IM. Width of band of ramenta. J. Width of diaphragm aperture. K. Ramenta. L. Height of perigone tube. M. Ridge on inner surface of perigone tube. N. Flange of ridge on inner surface of perigone tube. O. Fusion area of perigone tube ridges. P. Ovary (in female). Q. Column. S. Height of disk. T. Diameter of crest of disk. U. Depth of disk. WF. Stigmatic fascia (in female). WM. Longitudinal strip of tissue bearing anther (in male). Y. Vestigial anther (in female). Z. Anther (in male) [Based on the original in Bänziger and Hansen (1997), modified by H.Đ. Trần]

These populations were examined to determine if they formed a ‘cluster’ [defined by Bänziger et al. (2000) as individual blooms arising from the same host vine]. Sample open blooms, either fresh or not too badly deteriorated and still with recognisable colour patterns, were taken, examined and various parts were measured according to the protocol used by Bänziger and Hansen (1997) with slight modification (Fig. 2). Notes and photographs of different parts and their colour were taken. A Leica S6D microscope was used to examine a section through the ovarial chamber. Vouchers for both sexes from the Tuyen Lam Lake population (*Tran* et al*. 472* for the female, *Tran* et al*. 473* for the male) were deposited with the SGN and SING herbaria (herbarium acronyms follow Thiers, continuously updated), and vouchers for the Nam Ban population (*Q.Đ. Nguyễn and H.C. Nguyễn NB*-*001* for the female, *NB*-*002* for the male) have been lodged with the SGN herbarium. The identification of the host plant of *Sapria himalayana* was confirmed at the VNM herbarium.

## Results and discussion

### Distribution of *Sapria*

In Vietnam, *Sapria himalayana* is thus far known only on the Lang Biang Plateau, at Tuyền Lâm Lake, Cam Ly and Nam Ban Protection Forest. Outside of Vietnam, the known range of *Sapria himalayana* includes India and Thailand, where the forma *himalayana* (distinguished by yellow warts on blood-red perigone lobes) occurs in India (Assam), Myanmar and Thailand (Elliott 1992; Bendiksby et al. 2010) and the forma *albovinosa* Bänziger and Hansen (distinguished by white warts on wine-red perigone lobes) is documented from Phu Khiew Wildlife Sanctuary, Chaiyaphum Province, Thailand (Bänziger et al. 2000). This distribution is likely to be very incomplete because there are generally very few careful documentations of *Sapria* and their colour expression or variation.

The other two species of *Sapria* known include *S. poilanei*, so far known from the Cardamom mountains area in Cambodia and Chanthaburi in Thailand, and *S. ram* documented from Dawna-Bilauktaung, Phangnga and Suratthani in Thailand (Bänziger and Hansen 1997).

### Identification of the Lang Biang *Sapria*

**Fig. 3:**
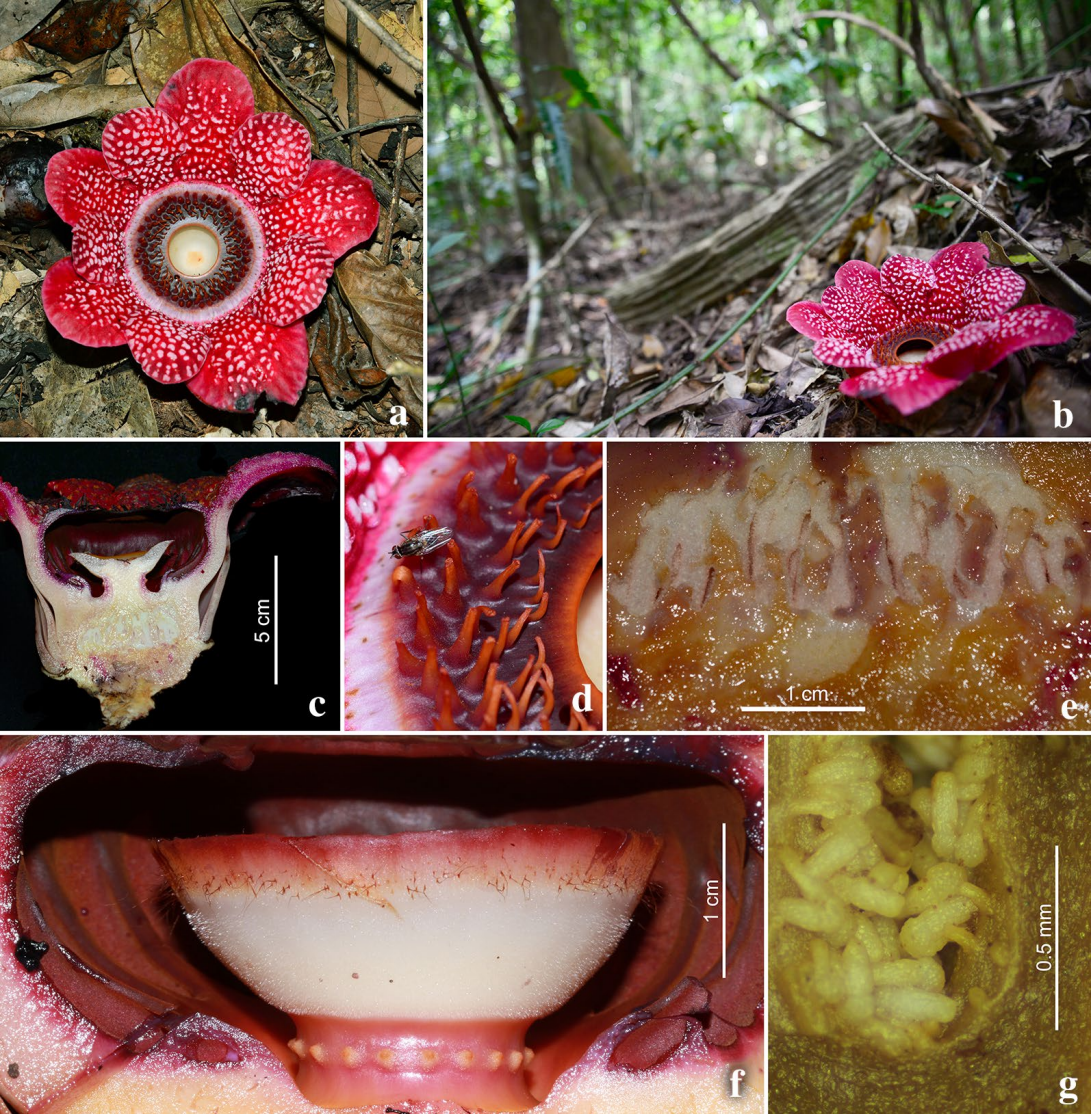
*Sapria himalayana* Griff. f. *albovinosa* Bänziger and Hansen, female flower. **a** Whole flower. **b** Habitat in natural forest at Tuyen Lam. **c** Longitudinal section of flower. **d** Ramenta on upper surface of diaphragm. **e** Cross section of ovary. **f** Lateral view of disk and upper part of column, with vestigial anthers (yellow structures in a ring below the disk) visible. **g** Ovules. Photos: H.Đ. Trần

**Fig. 4:**
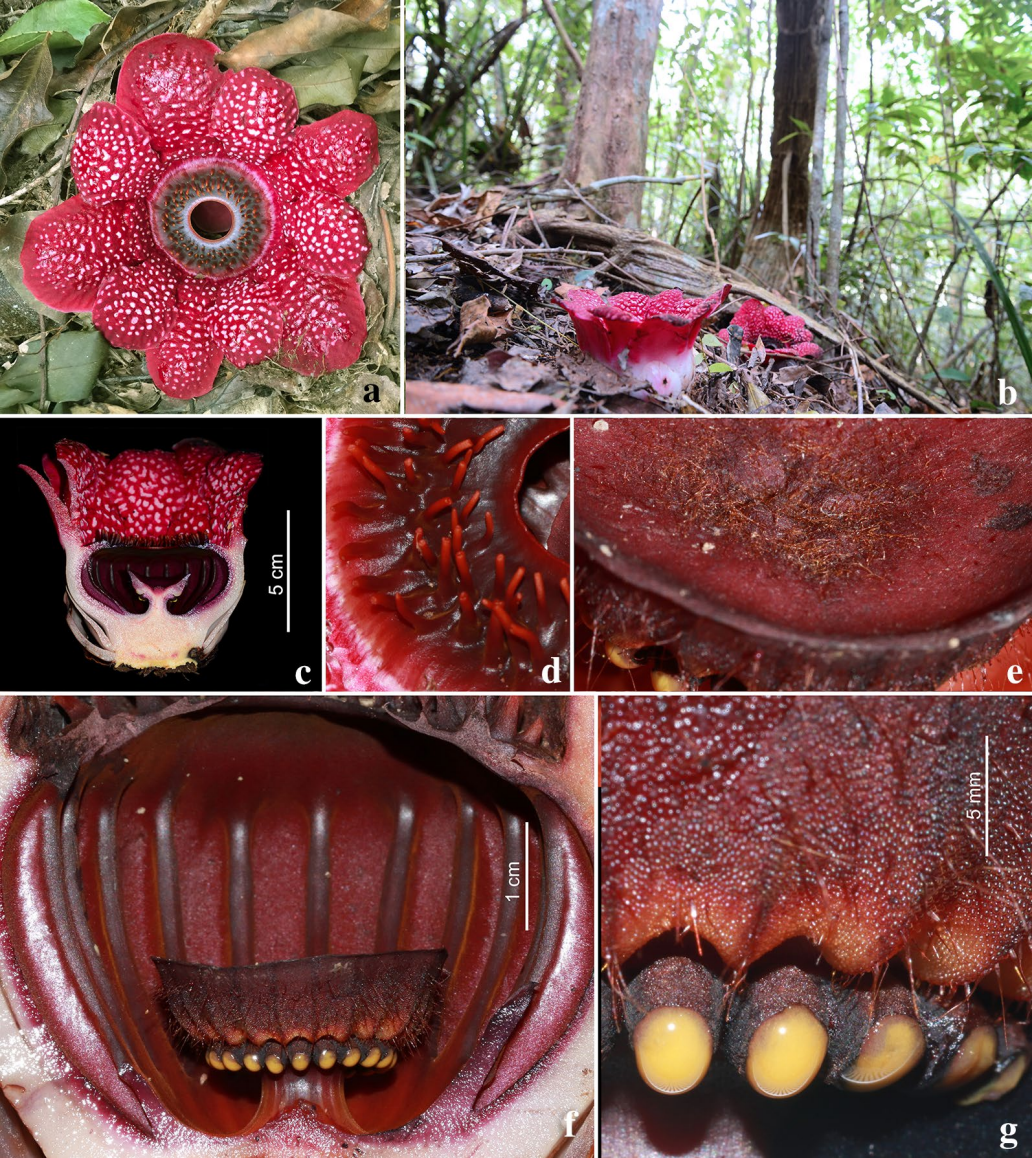
*Sapria himalayana* Griff. f. *albovinosa* Bänziger and Hansen male flower. **a** Whole flower. **b** Open flowers with more steeply held perigone lobes than in the female. **c** Longitudinal section of flower. **d** Ramenta on upper surface of diaphragm. **e** Part of upper disk surface. **f** Lateral view of column and disk, showing anthers (yellow structures) in a ring and longitudinal ridges on the inner surface of the perigone tube. **g** Anthers. Photos: H.Đ. Trần

By its size attributes, morphological details (Table 1) and colour patterns (Figs. 3, 4), we were able to identify the Vietnamese taxon as *Sapria himalayana* f. *albovinosa* according to the excellent key and attributes of fresh material provided by Bänziger and Hansen (1997) and Bänziger et al. (2000). Fresh open flowers of *Sapria himalayana* are distinctly larger (previously documented floral span 95–200 mm) than either *Sapria poilanei* (floral span 65–120 mm) or *Sapria ram* (floral span 55–110 mm) (Bänziger and Hansen 1997), and our Vietnamese taxon has a floral span of 110–165 mm, falling in the range for *S. himalayana*. The ramenta apices of the Vietnamese taxon are also bilobed or multilobed (especially in female specimens), or crateriform (i.e., with a shallow recess, especially in males). The aperture of the diaphragm is 14–21 mm, distinctly smaller than the disk of 51–61 mm diameter, as also documented by Bänziger and Hansen for *Sapria himalayana.* The disk is somewhat flat and thins out towards its periphery so that it does not have a distinctive “wall” between upper and lower surfaces, as noted by Bänziger and Hansen (1997) for *S. himalayana*; in contrast, the other two species have a pan- to bowl-shaped (female) or cup-shaped (male) disk with a distinct wall. Other morphological details (Table 1) are also consistent with those of *Sapria himalayana* as described by Bänziger and Hansen (1997). Comparison between these three *Sapria* species is summarized in Table 2.

**Table 1:** Measurements (in mm) of female and male flowers of *Sapria himalayana* f. *albovinosa* sampled on Lang Biang Plateau for the present study

Flowers	Code	♀ 1	♀ 2	♀ 3	♂ 1	♂ 2	♂ 3
Flower diameter	C	157–160	145–165	130–140	110–116	138–142	117–142
Lobe inclination from the vertical		60°–75°	30°–70°	55°–90°	0°–10°	30°–45°	10°–45°
Height of flower	HE	90	73	71	97	100	107
Circumference of perigone tube at lobe base	D	260	254	250	250	265	255
Height of perigone tube	L	34	32	32	31	37	38
Perigone lobe width/length (outer whorl)	B (E/F)	42–58/48–50	43.5–55/49–53	41–55/38–52	40–50/50–57	40–52/46–53	39–53/45–54
Perigone lobe width/length (inner whorl)	B (E/F)	32–34/43–46	31–37/38–41	32–34/41–46	36–37/44–51	33–38/40–50	31–37/39–44
Diameter of whole diaphragm	H	58–60	54–55	54–61	52–55	53–56	51–55
Width of diaphragm collar	I	21–24	20–21.5	18–21	16–18	18–19	16–17
Width of diaphragm aperture	J	14–16	14–15	19–20	18–20	19.5–20.5	20–21
Width of ramenta band	IM	14–15	12.5–15	11–14.5	9–12	12–13	11–12
Width of distal band	ID	6–7	6–7	5.5–7	5–6	5–6	4–5
Height of column	Q	20	18	16–17	16–17	17–19	Column and disk missing (eaten)
Diameter of column where narrowest		15	14	15	8	8	
Diameter of crest of disk	T	35–39	36–38	37–38	23–24	32–33	
Depth of disk	U	6	5	5–5.5	6–7	7–8	
Height of disk (to just above anthers)		11	10.5	10–11	9–10	8–9	
Width of stigmatic fascia/annular row of anthers	WF/WM	15	14	13.5	12–13	12	
Maximum diameter of stigmatic circle		32	32.5	32.5	–	–	
Maximum height of perigone tube ridges: width of its flange at that point	M:N	5:2	4.8:2.4	5.5:2.5	7	7	7
Height of cupule at flower base	CP	10–13	15–16.6	12–14.5	2–3	3–4.5	4–6
Outer whorl of basal bracts (length)	A	21–35	20–34	21–30	14–17	20–29	20–20
Inner whorl of basal bracts (length)	A	53–59	50–58	43–56	39–62	50–58	44–59

**Table 2:** Floral characters and known flowering period compared for three *Sapria* species

Species	*Sapria himalayana*	*Sapria poilanei*	*Sapria ram*
Flower diameter	95–200	65–120	55–110
Ramenta long (mm)	Up to 10	1–3(5)	5–5.5
Lobes colour	Blood-red	Wine-red	Wine-red
Dotted warts colour	Yellow (or white)	White (sometimes pale pink)	White (sometimes pale pink)
Aperture diameter (mm)	18–37	15–25	13–29
Female disk shape	Bowl-shaped	Bowl-shaped	Pan-shaped
Female disk’s crest diameter (mm)	35–39	9–18	22–28
Male disk shape	Bowl-shaped	Cup-shaped	Cup-shaped
Male disk’s crest diameter (mm)	23–33	12–15.5	14–18
Flowering period	November–February	November–March	November–March

**Fig. 5:**
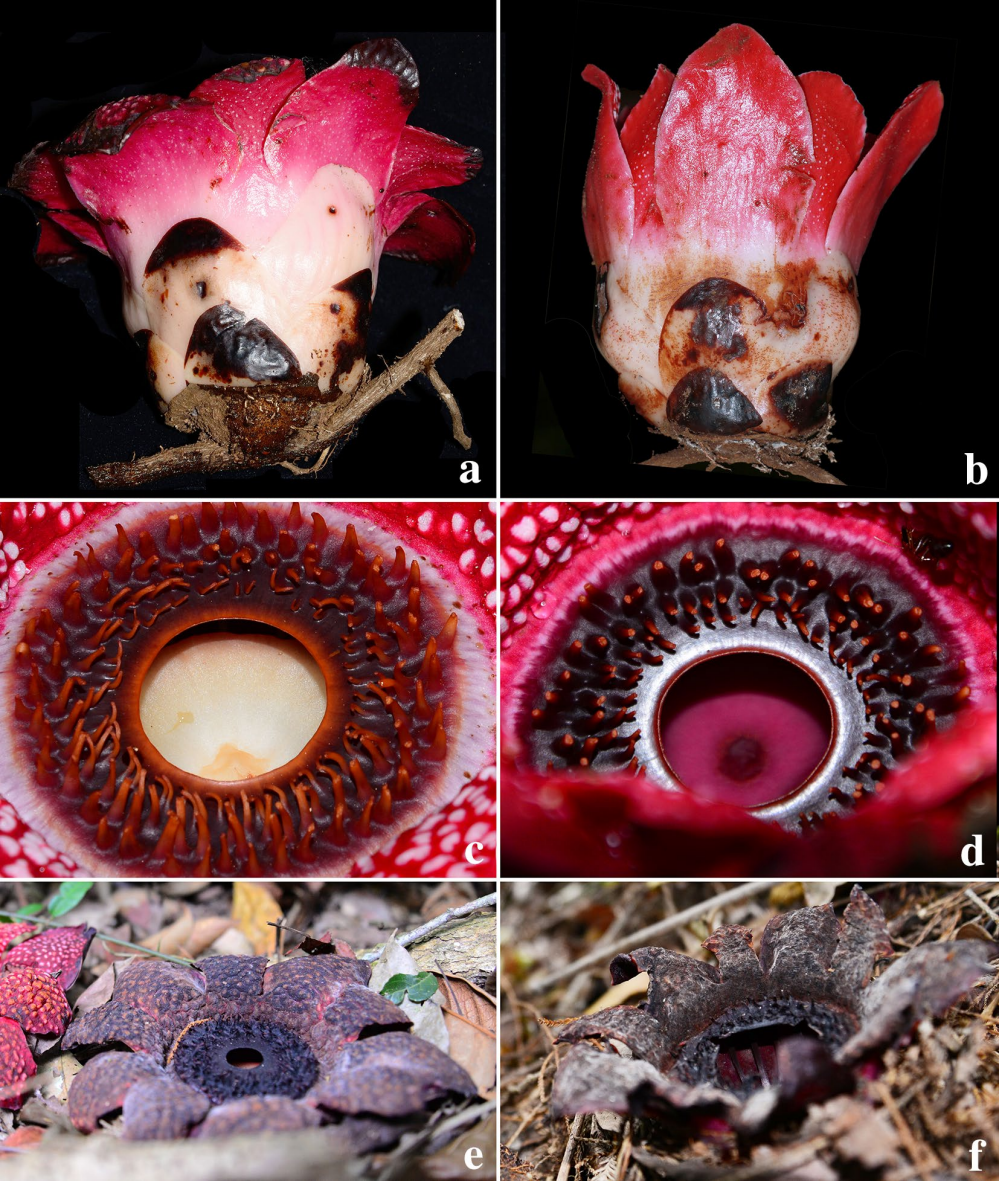
*Sapria himalayana* Griff. f. *albovinosa* Bänziger and Hansen. **a** Female flower with higher cupule and wider spreading perigone lobes. **b** Male flower with lower cupule and more steeply held perigone lobes. **c** Diaphragm and aperture of female flower with a pale disk. **d** Diaphragm and aperture of male flower, with reddish disk. **e** Post-anthesis female flower. **f** Post-anthesis male flower. Photos: H.Đ. Trần

The colour patterns of the Vietnam taxon (Figs. 3, 4, 5 and 7) are those documented for *Sapria himalayana* f. *albovinosa*. The warts are white against the distinctive wine-red colour of the perigone lobes as described for this form.

### Description of the Lang Biang *Sapria*

In the following description, the key morphological parts are provided with abbreviations in brackets that correspond to Fig. 2.

Holoparasitic plant on *Tetrastigma laoticum* roots. Flower buds c. 8 cm in diameter near to opening stage. Cupule (CP) 10–16.6 mm high in female, 2–6 mm high in male. Bracts (A) of outer whorl 5, (14–)20–35 mm long; those of inner whorl 5, (39–)43–62 mm long. Diameter (C) × height (HE) of fully open flowers: 130–165 × 71–90 mm in female, 110–142 × 97–107 mm in male. Perigone lobes 10 (inner whorl 5 and outer whorl 5); female lobes when fully open (30–)60° to 90° from the vertical plane through the floral centre; male lobes when fully open 0° to 30(–45)° from the vertical plane through the floral centre; outer lobes ovate, length (F) 38–57 × width (E) 39–58 mm, red with white warts adaxially, with white dots abaxially, glabrous; inner lobes spathulate, 38–51 × 31–38 mm, with white warts adaxially, with white dots abaxially, glabrous. Diaphragm 51–61 mm in diameter (H); 14–21 mm across aperture (J); collar width (I) 16–24 mm; basal band (IB) white with pink radial stripes, turning red then purple-black with age; ramenta band (IM) 9–15 mm wide, reddish black, turning purple-black with age, ramenta (K) mostly bi- or multilobed in females and occasionally in males, mostly crateriform in males and occasionally in females, 5–9 mm long, wine-red at base, turning dark orange to dark red towards their apex, blackening with age; distal band (ID) 4–7 mm wide, dark red turning black with age. Perigone tube circumference (D) around lobe bases 254–265 mm, height (L) 31–38 mm. Central column (Q) dimorphic between sexes: female column 16–20 mm high, 14–15 mm in diameter at narrowest point, disk bowl-shaped, upper surface whitish cream, margin reddish, crest of disk (T) 35–39 mm in diameter, depth of disk (U) 5–6 mm, height of disk (S) to just above vestigial anthers 10–11 mm, stigmatic fascia (WF) 13.5–15 mm wide, sparsely bristly; male column 16–19 mm high, c. 8 mm in diameter at narrowest point, disk bowl-shaped, upper surface wine-red, margin dark red, crest of disk (T) 23–33 mm in diameter, depth of disk (U) 6–8 mm, height of disk to just above anthers 8–10 mm, longitudinal strip of tissue bearing anther (WM) 12–13 mm long, hairy. Anthers (Z) 20, set in a ring, each 2.5 mm wide, filaments 2.0–2.3 mm long. Ovary (P) inferior, 36–38 × 14–20 mm, cavities irregular and bearing many ovules.

### Additional features of sexual dimorphism from the Lang Biang observations

Bänziger and Hansen (1997) have summarised the distinction between females and males by differences mainly in the column (stalk and disk) form and structure, the colour of the upper disk surface, the form and dimensions of radial ridges on the inner surface of the perigone tube, and the occurrence of ovarial chambers in an expanded base below the column in the female. The *Sapria himalayana* f. *albovinosa* populations in our study area also conformed in these characteristics.

Additionally, we have observed several more attributes that help in the distinction between the sexes in *Sapria himalayana* f. *albovinosa*. For the Lang Biang populations examined, all males consistently had more steeply held perigone lobes (spreading 0–45° from the vertical) than females, in which the lobes were more spread out at wider angles (spreading 30–90° from the vertical) in fully open flowers (Figs. 3c, 4c, Table 1). Male flowers that were past anthesis and beginning to appear discoloured were also examined, and were found to have similarly steeply held perigone lobes, indicating that this was indeed the full extent of floral opening in males (Fig. 5e, f). We remain uncertain if biomechanical reasons linked to structural variation have caused these differences.

The basal cupules of male and female flowers were also different. Males have a much lower cupule (2–6 mm high) than females (10–16.6 mm high) (see Table 1, Fig. 5). This difference appears to be a significant and easy character for quick determination of the sex even when the flower is in the advanced bud stage, i.e., when the bud begins to reveal the apex of the paler colored developing perigone lobes (and before the other main characters can be assessed), and so might be of potential importance in field investigations that require special preparations for sampling or observation of the different sexes.

### Some natural history aspects

**Fig. 6:**
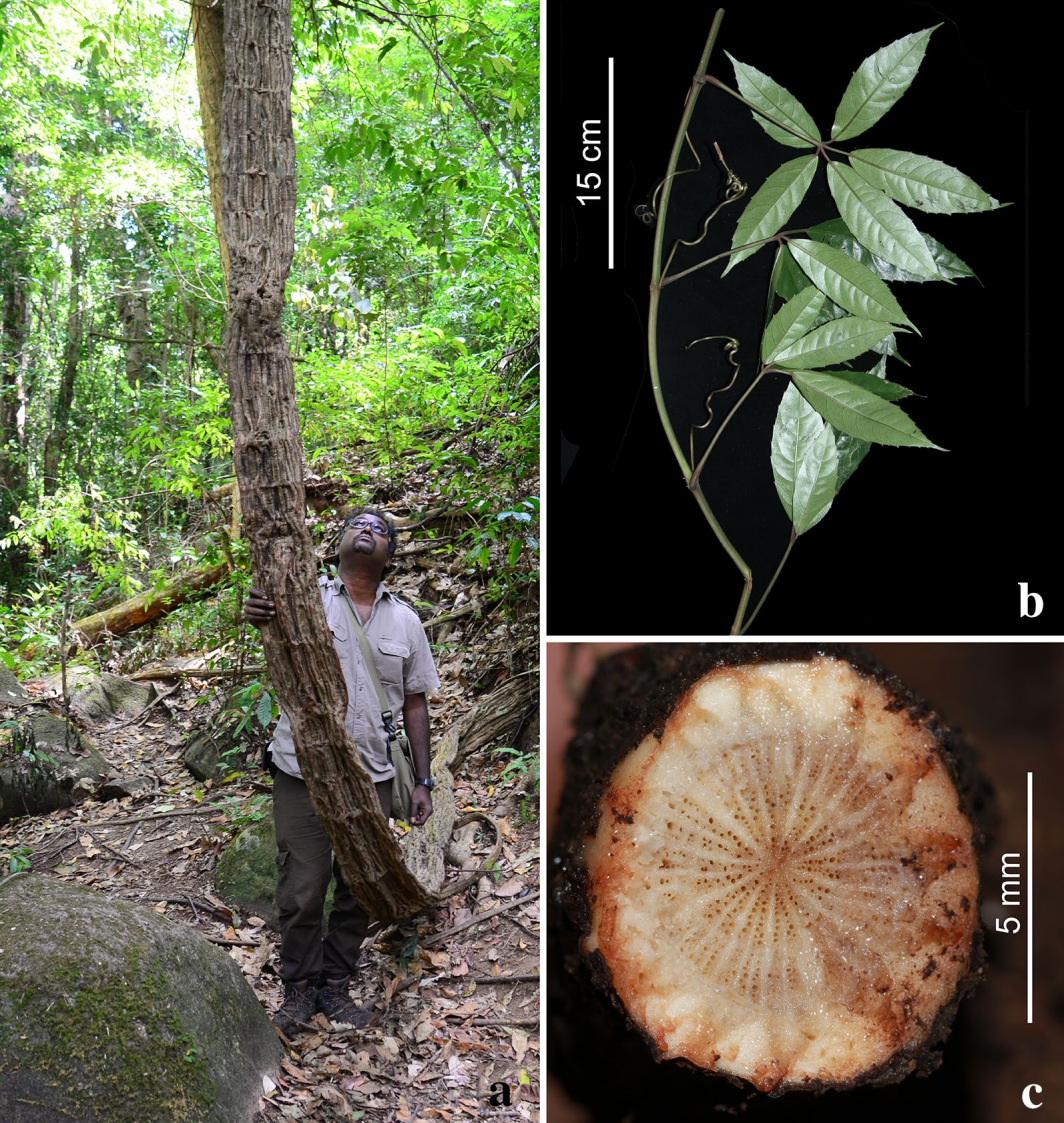
*Tetrastigma laoticum*, the host vine of *Sapria himalayana*. **a** Mature climbing stem with flat structure due to extended secondary thickening of the original stem axis in opposite directions; demonstrated by Parusuraman Athen. **b** Leaves with tendrils arising from petiole bases. **c** Cross section of root near emergence of *S. himalayana* flower. Photos: H.Đ. Trần

**Fig. 7:**
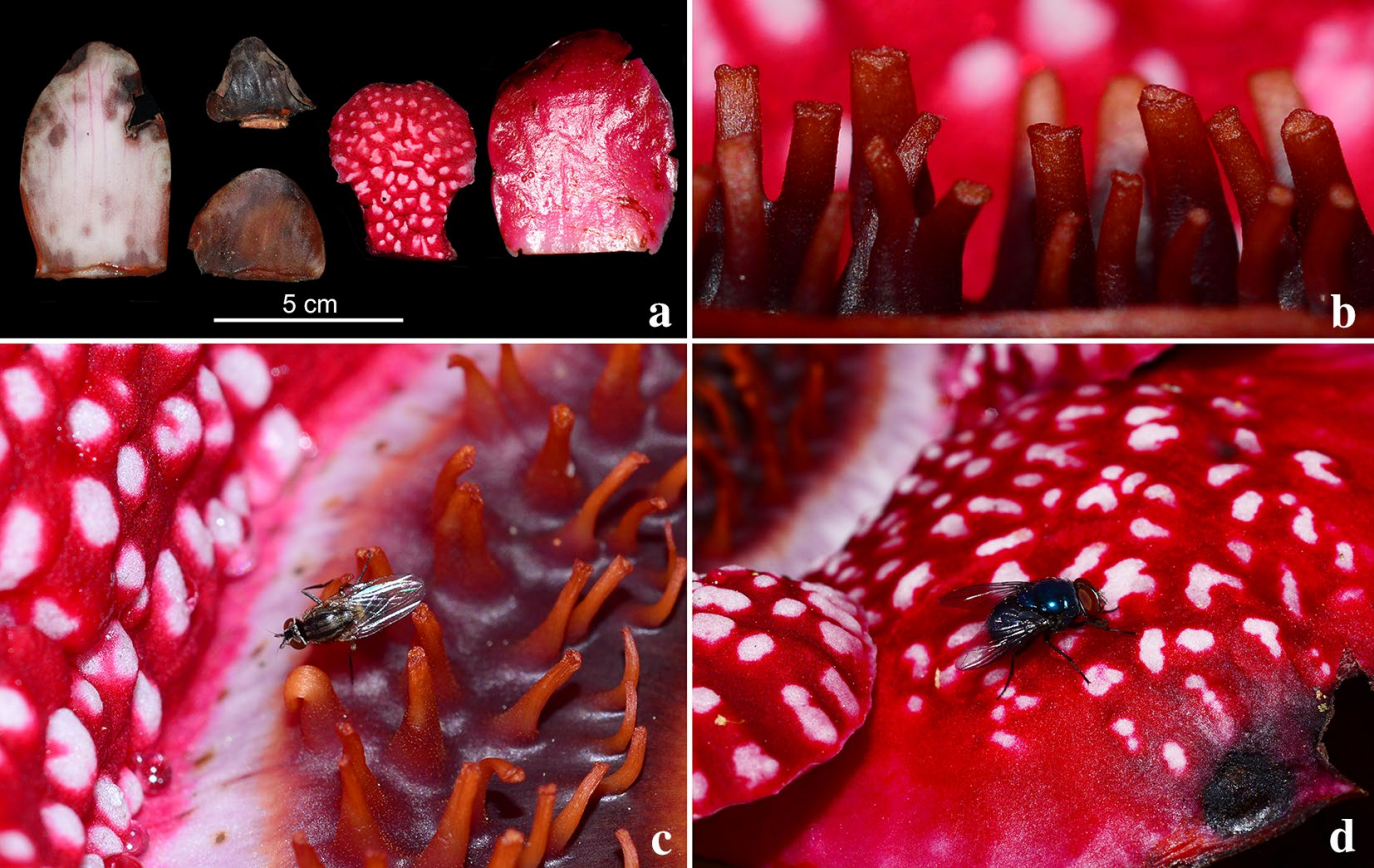
*Sapria himalayana* Griff. f. *albovinosa* Bänziger and Hansen. **a** Bracts (darker) and perigone lobes (red, patterned) in male flower. **b** Ramenta in male flower. **c** Detail of perigone lobe warts and Stratiomyidae fly. **d** Calliphoridae fly on *Sapria* flower. Photos: H.Đ. Trần

The host plant (Fig. 6) was identified as the liana *Tetrastigma laoticum* Gagnep. (Vitaceae). Although the host plant populations at the Lang Biang Plateau sites were dense, we were unsure of their genetic variation and relationships, as lianas often drape onto the forest floor and can vegetatively generate new individuals. More detailed studies of the host vine relationships of *Rafflesia* by Pelser et al. (2016) have demonstrated that the more common species of *Rafflesia* can have multiple host vines, so that host-parasite specificity is not as great as previously supposed. In other words, the potentially clonal nature of neighbouring host lianas was uncertain. It was also not known if the phenological behaviour of the host lianas, e.g., if a state of flowering had any possible effects on their predisposition to infection by and establishment of the parasite. From our observations, male and female flowers can be mixed together in the same cluster from one individual liana. Flowers are red when open, turning black as they senesce. We have not specially studied the pollinators but were able to note that flies belonging to the Calliphoridae and Stratiomyidae families visited open flowers (Fig. 7c, d). The pollination biology of *Sapria* is similar to that of *Rafflesia* in that pollen from the anther is deposited on the visiting flies, which is then carried to other flowers (Heide-Jørgensen 2008).

There is currently a dearth of information on seed development, seed dispersal, germination, as well as ecology in terms of infection and establishment for *Sapria* species. Better observations of these aspects will only be possible where a good number of populations are present, allowing comparative work, within secure sites where disturbance is likely to be absent or minimal, such as at the presently known Vietnamese sites. Also, the security and commonness of the parasite on site would enable possibilities for tagging and longer-term observations to be carried out, such as phenology and other ecological attributes, and plans are being formed by our research group for suitable studies on *Sapria himalayana* f. *albovinosa*.

### Conservation management

Both known *Sapria* sites on the Lang Biang Plateau are within relatively undisturbed broadleaf evergreen montane forest with *Acer* (Aceraceae), Fagaceae trees and *Tetrastigma* (Vitaceae) lianas commonly occurring. Recent studies of genetic populations of Rafflesiaceae have indicated that loss of a single infected host vine could result in large genetic losses of the parasite (Barkman et al. 2017), thus conservation activities should also focus on the conservation of host vine populations.

Whereas the Nam Ban site is itself a protection forest, the Tuyền Lâm site is within the Lâm Viên Protection Forest where only light tourism activities (e.g., forest trekking and interpretation) are permitted.

Following our investigations, the Southern Institute of Ecology has alerted authorities of the Lâm Đồng Province, under which jurisdiction the sites are administered, for the sites to be specially demarcated as Protected Research Reserves. This special status will no doubt enhance further careful use of designated parts of the Tuyền Lâm site for nature tourism, as well as maintain the integrity of the other strictly protected parts that will, among other things, contribute to the conduct of our longer-term studies.
